# A Human Osteocyte Cell Line Model for Studying *Staphylococcus aureus* Persistence in Osteomyelitis

**DOI:** 10.3389/fcimb.2021.781022

**Published:** 2021-11-03

**Authors:** Nicholas J. Gunn, Anja R. Zelmer, Stephen P. Kidd, Lucian B. Solomon, Eugene Roscioli, Dongqing Yang, Gerald J. Atkins

**Affiliations:** ^1^ Centre for Orthopaedic & Trauma Research, Faculty of Health and Medical Sciences, University of Adelaide, Adelaide, SA, Australia; ^2^ Australian Centre for Antimicrobial Resistance Ecology, University of Adelaide, Adelaide, SA, Australia; ^3^ Research Centre for Infectious Disease, School of Biological Science, University of Adelaide, Adelaide, SA, Australia; ^4^ Department of Orthopaedics and Trauma, Royal Adelaide Hospital, Adelaide, SA, Australia; ^5^ Department of Thoracic Medicine, Royal Adelaide Hospital, Adelaide, SA, Australia; ^6^ Department of Medicine, Faculty of Health and Medical Sciences, University of Adelaide, Adelaide, SA, Australia

**Keywords:** osteomyelitis, *Staphylococcus aureus*, intracellular infection, human, osteocyte, chronic infection, small colony variant (SCV)

## Abstract

Infectious osteomyelitis associated with periprosthetic joint infections is often recalcitrant to treatment and has a high rate of recurrence. In the case of *Staphylococcus aureus*, the most common pathogen in all forms of osteomyelitis, this may be attributed in part to residual intracellular infection of host cells, yet this is not generally considered in the treatment strategy. Osteocytes represent a unique cell type in this context due to their abundance, their formation of a syncytium throughout the bone that could facilitate bacterial spread and their relative inaccessibility to professional immune cells. As such, there is potential value in studying the host-pathogen interactions in the context of this cell type in a replicable and scalable *in vitro* model. Here, we examined the utility of the human osteosarcoma cell line SaOS2 differentiated to an osteocyte-like stage (SaOS2-OY) as an intracellular infection model for *S. aureus*. We demonstrate that *S. aureus* is capable of generating stable intracellular infections in SaOS2-OY cells but not in undifferentiated, osteoblast-like SaOS2 cells (SaOS2-OB). In SaOS2-OY cells, *S. aureus* transitioned towards a quasi-dormant small colony variant (SCV) growth phenotype over a 15-day post-infection period. The infected cells exhibited changes in the expression of key immunomodulatory mediators that are consistent with the infection response of primary osteocytes. Thus, SaOS2-OY is an appropriate cell line model that may be predictive of the interactions between *S. aureus* and human osteocytes, and this will be useful for studying mechanisms of persistence and for testing the efficacy of potential antimicrobial strategies.

## Introduction

Infectious osteomyelitis, also known as deep bone infection, is a serious clinical feature of periprosthetic joint infection (PJI) associated with chronic disease. PJI occurs in around 1-4% of hip and knee arthroplasties, although this incidence is likely underreported (Tande and Patel, 2014; Mitchell et al., 2017). This prevalence is concerning due to the high associated morbidity and mortality, with hip and knee PJI having a 5-year mortality of 21.12% and 21.64%, respectively (Lum et al., 2018; Natsuhara et al., 2019). In addition, the increased cost of treatment places a significant burden on healthcare systems, which will be exacerbated by the increasing demand for joint replacement surgery. Staphylococci are the most common causative pathogens of PJI and infectious osteomyelitis in other clinical scenarios, and *Staphylococcus (S.) aureus* is the most common organism (Tande and Patel, 2014).

Intracellular *S. aureus* has been found to be causative of antibiotic-resistant, recurrent infections in various host cell types (Plouin-Gaudon et al., 2006; Zautner et al., 2010). As few as one hundred colony forming units (CFU) of intra-osteoblastic *S. aureus* was capable of inducing acute bone infections in a rat open fracture model, providing strong evidence for the intracellular niche constituting a reservoir for infection recurrence (Hamza et al., 2013). Our group described the human osteocyte as a potential reservoir for *S. aureus* during chronic PJI (Yang et al., 2018). In that study, osteocytes were demonstrated to robustly respond to *S. aureus*, at least at a multiplicity of infection (MOI) of 100, through innate and adaptive immune mediator expression, among a multitude of cellular pathways, using multiple experimental models including human primary osteocyte-like cells, human bone *ex vivo* cultures and transcriptome analyses of post-surgical human PJI bone specimens. The intra-osteocytic bacteria transitioned to the quasi-dormant small colony variant (SCV) phenotype and importantly for the longevity of these infections, induced minimal cytotoxic effects on the host cell. This scenario has significant implications as a discrete phenomenon that can be modelled to inform assessments of sustained infection.

Quiescent bacterial growth phenotypes are well suited to intracellular persistence as their reduced expression of virulence factors reduces immunogenicity, averting acute inflammatory responses. However, quiescent bacteria remain capable of reverting to an active growth phenotype and thus initiating a new acute infection following intracellular escape (Tuchscherr et al., 2011). As bactericidal antibiotics almost all function through blocking various active biosynthetic processes (e.g. DNA, RNA, protein or cell wall synthesis), by reducing metabolic activity and replication, quasi-dormant bacterial forms are capable of tolerating antibiotic challenge. In addition, host cells can protect resident bacteria from antibiotics through poor membrane penetrance and uptake, antibiotic degradation and efflux, the sequestration of antibiotics and the bacteria into separate subcellular compartments and the induction of a transition towards quasi-dormant growth phenotypes (Vesga et al., 1996; Proctor et al., 2006; Nguyen et al., 2009; Bongers et al., 2019). For example, the low pH of phagolysosomes has been shown to increase *S. aureus* resistance to some antibiotics that are used clinically to treat these infections - including gentamicin, rifampicin and clindamycin - with this effect mediated through changes to the bacteria or the antibiotic itself (Lam and Mathison, 1983; Baudoux et al., 2007; Thomas et al., 2012). In addition to the reduced efficacy of many treatments, the intracellular niche is inaccessible to the humoral immune response and so any immune response to these bacteria needs to be mediated by the host cell or cell-mediated immunity.

Osteocytes are encased within the bone lacunocanalicular network. This environment limits cell-mediated immune surveillance for osteocytes relative to other cell types. In addition, osteocytes are a long-lived cell population, potentially living for decades *in vivo* (Prideaux et al., 2016). All of these features arguably make osteocytes the perfect harbour for intracellular, persistent pathogens. It is noteworthy, however, that we have found PJI to be associated with increased osteocyte-mediated bone matrix degradation (Ormsby et al., 2021), which could facilitate the clearance of osteocytes harbouring dormant bacteria by both the destruction of the host cell and the facilitation of access to the cell-mediated immune system, although these possibilities remain to be explored.

To date, a human cell line model of intra-osteocytic infection has not been described. We previously demonstrated that the SaOS2 cell line is capable of differentiating into an osteocyte-like state (Prideaux et al., 2014). In this study, we characterised differentiated osteocyte-like SaOS2 cells, here designated SaOS2-OY, as a human cell line model for intra-osteocytic *S. aureus* infection.

## Materials And Methods

### Cell Culture

SaOS2 cells were maintained in growth media consisting of αMEM (Gibco, NY, USA) supplemented with 10% v/v foetal calf serum (FCS), 50 μg/ml ascorbate 2-phosphate and standard tissue culture additives (10 mM HEPES, 2 mM L-Glutamine, penicillin/streptomycin each 1 unit/ml, Thermo-Fisher, VIC, Australia) at 37°C/5% CO_2_ (Prideaux et al., 2014). To achieve an osteocyte-like phenotype, SaOS2 cells were switched to differentiation media at confluence, consisting of αMEM supplemented with 5% v/v FCS, 50 μg/ml ascorbate 2-phosphate, standard tissue culture additives and 1.8 mM potassium di-hydrogen phosphate (Sigma, St Louis, USA) and then cultured at 37°C/5% CO_2_ for 28 days (Prideaux et al., 2014). For experimentation, cells were seeded at a density of 2 x10^4^ cells/cm^2^ and either grown to confluence without differentiation, representing an osteoblast-like (OB) state (here designated SaOS2-OB) or differentiated for 28 days (SaOS2-OY). Based on microscopy and haemocytometry, SaOS2-OB reached a cell density of ~2.5 x10^4^ cells/cm^2^ at confluence and the cell number increased to ~1 x 10^5^ cells/cm^2^ for SaOS2-OY after the 28-day differentiation period.

### Bacterial Culture and Establishment of Host Cell Infection

All *S. aureus* strains tested, including the methicillin-resistant (MRSA) strain WCH-SK2 (Bui et al., 2015), clinical osteomyelitis *S. aureus* strains isolated at the Royal Adelaide Hospital from patients undergoing distal amputation for diabetic foot infection, and RN6390 (Qazi et al., 2004), were grown in nutrient broth (NB), consisting of 5g/l NaCl, 3g/l beef extract and 10g/l peptone (Chem-Supply, SA, Australia), on a 37°C/200rpm rocking platform. The bacterial viable cell concentration for each experiment was estimated from a colony forming unit (CFU)/ml *vs* OD_600nm_ standard curve, then validated by plating dilutions on NB agar, consisting of NB with 1.5% w/v bacteriological agar (Sigma-Aldrich).

For infections, bacteria were pelleted at 10000 × g for 10 min, then resuspended in sterile PBS to the target density for the various multiplicities of infection (MOI) according to the requirements of the experiment in question. Culture media was removed and the cells were washed twice with PBS before the bacterial suspension or PBS control was added. Bacteria were incubated with host cells at 37°C for 2h, followed by two further washes and incubation at 37°C with 10 µg/ml lysostaphin (AMBI Products LLC, Lawrence, NY, USA) in antibiotic-free media for 2h to eliminate extracellular bacteria. Following the removal of extracellular bacteria, fresh differentiation media with or without antibiotics, depending on the specific experiment, was added to the cells. Culture supernatants were verified to be sterile by agar plating at 24h post-infection. Media were changed every 7 days during the experimental period (Yang et al., 2018).

### Fluorescent Confocal Microscopy

Both SaOS2-OB and SaOS2-OY cultures were prepared on glass chamber slides (ibidi GmbH, Martinsried, Germany) and infected with the GFP expressing *S. aureus* strain RN6390 (Qazi et al., 2004). Prior to imaging, infected cells were stained with Lysoview 540 (Biotium, CA, USA) and SiR-actin (Spirochrome, Switzerland) for 1h at 37°C for the visualisation of lysosomal bodies and cytoskeletal components F-actin, respectively. The invasion of *S. aureus* into host cells was monitored using an Olympus FV3000 confocal microscope (Olympus, Tokyo, Japan).

### Measurements of Host Cell Viability, Intracellular Bacterial Number and Growth Phenotype

SaOS2-OB and SaOS2-OY cultures seeded in 48-well tissue culture plates were infected as described for 24h and processed for the measurement of host cell viability using the lactate dehydrogenase (LDH) enzyme activity assay, as per manufacturer’s instructions (Sigma-Aldrich). For the measurement of intracellular bacterial number and growth phenotype, replicate infected cultures were lysed at various time points post-infection with 0.2% v/v Triton X-100 (Sigma-Aldrich) in water for 20 min at 37°C. Serially diluted cell lysates were plated on NB agar and incubated up to 120h at 37°C in a 5% CO_2_ humidified incubator. For each time point, CFU were recorded after 24h incubation and at 120h, with the difference between these values, i.e. late appearing CFU, being recorded as ‘slow growth’. A comparison of colony phenotype based on size was recorded at 24h culture for each time point, with the threshold of 1mm to determine ‘large’ or ‘small’ colonies.

### Measurement of Host Cell Gene Expressions by RT-PCR

For the quantification of gene expression, total RNA was isolated using Trizol reagent (Life Technologies, NY, USA) and complementary DNA (cDNA) templates were prepared using the iScript RT kit (BioRad, CA, USA), as per manufacturer’s instructions. Real-time RT-PCR reactions were then performed to determine the mRNA levels of target genes using RT2 SYBR Green Fluor qPCR Mastermix (Qiagen, Limburg, Netherlands) on a CFX Connect Real Time PCR System (BioRad). The sequences of the oligonucleotide primer sets targeting each gene are listed in **Supplementary Table 1**. Gene expression relative to the level of *ACTB* mRNA was calculated using the 2^−ΔCt^ method.

### Transmission Electron Microscopy (TEM)

To visualise infected SaOS2-OY cells by TEM, cells were cultured in 75cm^2^ cell culture flasks and exposed to *S. aureus* strain WCH-SK2, at MOI 100. Infected cells were sampled at 2h and 24h post-infection, by immediately placing TEM fixative (1.25% v/v glutaraldehyde, 4% w/v sucrose and 4% w/v paraformaldehyde in PBS) into cultures for 24h at 37°C. Cells were then demineralised by replacing the TEM fixative with 20ml of Osteosoft™ solution (Sigma-Aldrich). Each of the cell layer samples was removed by scraping and further processed for TEM imaging, as previously described (Yang et al., 2018).

## Results

### Osteogenic Differentiation Confers Enhanced Suppression of Intracellular Infection

**Figure 1:**
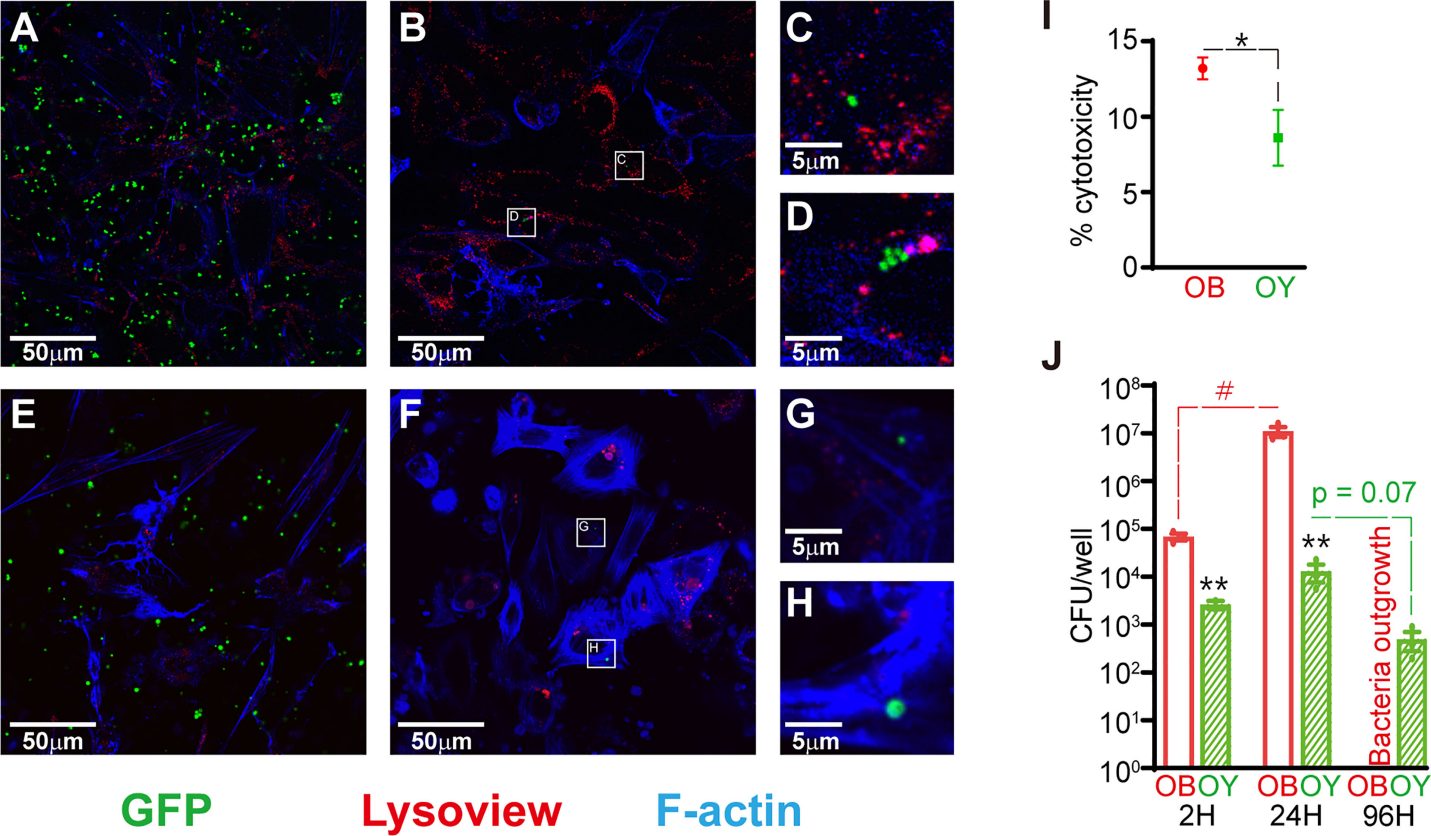
Infection dynamics in SaOS2-OB and SaOS2-OY cells. **(A–H)** Fluorescent confocal images taken from live samples with the GFP-expressing *S. aureus* strain RN6390 (green), Lysoview 540 which stains lysosomal bodies (red) and SiR-actin which stains F-actin (blue). Images were taken 2h following inoculation in SaOS2-OB **(A)** and SaOS2-OY **(E)**, as well as after 24h in SaOS2-OB **(B)** and SaOS2-OY **(F)**. Higher magnification images of intracellular bacteria represented by white squares in **(B, F)** are shown in **(C, D, G, H)**, respectively. **(I)** the % cytotoxicity of 24h of WCH-SK2 infection of either SaOS2-OB or SaOS2-OY was measured using an LDH assay at an MOI of 1. **(J)** Intracellular infection dynamics were determined using CFU counts recovered from either SaOS2-OB or SaOS2-OY cell lysates infected with WCH-SK2 (MOI of 1). The cells were cultured within antibiotic-free media following 2h of lysostaphin treatment immediately post-infection to kill bacteria that failed to invade into the intracellular niche. Data shown are means of biological triplicates ± standard error of the mean. Significant differences between cell types are indicated by *p < 0.05 and **p < 0.01, respectively. Significant change as a function of time is indicated by ^#^ *p* < 0.05.

In order to determine if the osteocyte-like state affected the host-pathogen interaction, initial experiments were performed to compare SaOS2 in either an undifferentiated, osteoblast-like (SaOS2-OB) or osteocyte-like (SaOS2-OY) differentiation state. In SaOS2-OB, intracellular bacteria were commonly observed in clusters after 24h, consistent with intracellular replication (**Figures 1B–D**). Bacteria within SaOS2-OY (**Figures 1F–H**) were not seen in clusters across the same time frame. Occasional bacteria in SaOS2-OY cells appeared of abnormal size (e.g. **Figure 1H**), perhaps representing cells undergoing abnormal fission and transitioning to an SCV phenotype (Yang et al., 2018). Interestingly, bacteria were rarely observed interacting with lysosomal bodies, especially in SaOS2-OY cells. Additionally, SaOS2-OB cells demonstrated an elevated number of lysosomal bodies, evident both at 2h following exposure to bacteria (**Figures 1A, E** **)** and within uninfected controls (**Supplemental Figure 1**).

SaOS2-OB were more susceptible to cytotoxicity than SaOS2-OY in response to 24h of infection (**Figure 1I**). Following exposure of the two cell types to *S. aureus* at a similar low MOI of approximately 1, SaOS2-OY took up significantly fewer of the inoculating bacteria (6.5% v. 67.8% for SaOS2-OY and SaOS2-OB, respectively, *p* < 0.01) **(** **Figure 1J**). The culturable bacteria within SaOS2-OB replicated more readily and between 1 and 4 days post-infection (DPI) escaped into the media. SaOS2-OY across this same time period suppressed the replication of culturable bacteria resulting in a downward trend in CFU/well over time with no bacteria culturable within the media at 4 DPI.

### Intracellular Persistence of *S. aureus* Within SaOS2-OY

**Figure 2:**
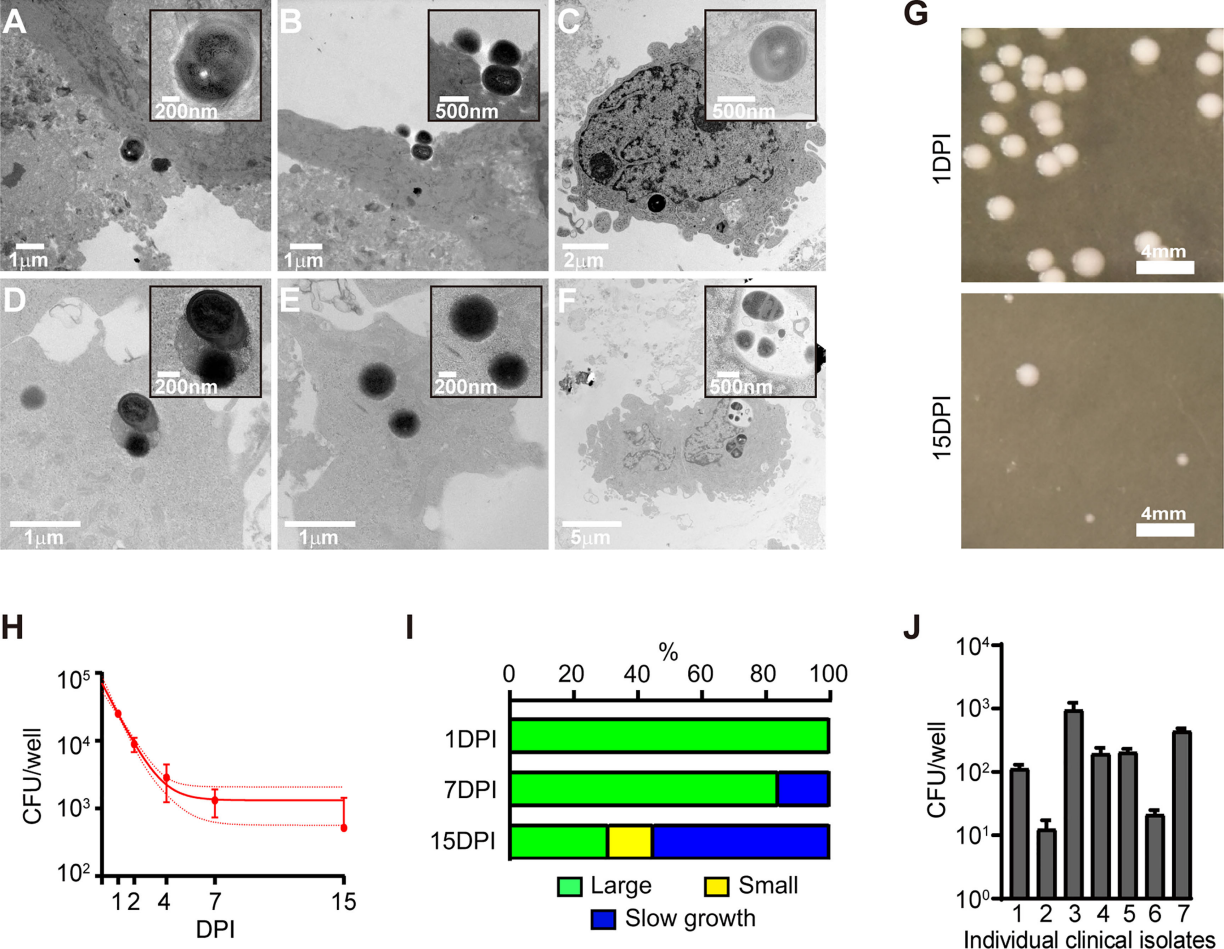
Characterisation of the intracellular infection of SaOS2-OY. **(A–F)** Transmission electron microscopy images of SaOS2-OY either following 2h **(A, B)** or 24h **(C–F)** of exposure to WCH-SK2. **(G)** Representative images of colonies grown after 24h of plate incubation from the infection presented in **(H)**. **(H)** Infection dynamics of WCH-SK2 within the lysate of SaOS2-OY cultured within lysostaphin enriched media. Data shown are means ± 95% confidence intervals. **(I)** Distribution of colony morphology classifications across the duration of the infection presented in H. **(J)** Bacterial burden measured after 24h of intracellular infection with a range of *S. aureus* clinical osteomyelitis isolates, all assayed at a confirmed MOI of 0.5. DPI, Days post-infection; CFU, Colony forming units.

As it was apparent that osteogenic differentiation modified the host-bacteria interaction, we sought to characterise the infection dynamics. Following a 2h exposure of SaOS2-OY to WCH-SK2, bacteria were observed co-localised with the plasma membrane and undergoing internalisation (**Figures 2A, B** **)**. Intact intracellular bacteria were observed after 24h within structures consistent with those commonly reported as autophagosomes or phagosomes, i.e. vacuoles containing a large electron lucent gap surrounding the entrapped bacteria (**Figure 2F**). Bacterial cells were also observed either free within the cytoplasm or within less well-defined vacuoles with either intact (**Figure 2C**), semi-compromised (**Figure 2D**) or fully compromised (**Figure 2E**) cell walls. Additionally, cells undergoing abnormal division evidenced by small electron lucent puncta at the division plane and/or asymmetrical division were also observed (**Figures 2A, F** **)**, consistent with reports of cell stress and SCV generation (Li et al., 2011; Onyango et al., 2012; Loss et al., 2019).

Following infection of SaOS2-OY cells with WCH-SK2, host cell lysates were cultured on nutrient agar plates at multiple time points over a 15d period (**Figures 2G, H** **)**. The total CFU number decreased with time (**Figure 2H**), and the colony morphology transitioned from a ‘large’ growth phenotype (defined here as >1 mm in colony diameter after 24h of plate incubation) to predominantly ‘small’ colonies (defined here as <1 mm in colony diameter after 24h of plate incubation) (**Figures 2G, I** **)**. Additionally at both 7 and 15 DPI a proportion of ‘slow growth’ colonies arose.

Exposure of this cell model to a range of clinical osteomyelitis isolates of *S. aureus* resulted in the consistent establishment of intracellular infections that were culturable at 1 DPI, albeit with differing intracellular yields (**Figure 2J**).

### SaOS2-OY Displays a Proinflammatory Response to WCH-SK2 Infection

We next characterised the impact of intracellular infection on host gene expression. Based on our findings with human primary osteocyte-like cells (Yang et al., 2018; Ormsby et al., 2021), we selected a panel of genes representing the innate immune response and bone remodeling or degradation markers, and measured their expression change relative to uninfected cells as a function of MOI and time post-infection.

**Figure 3:**
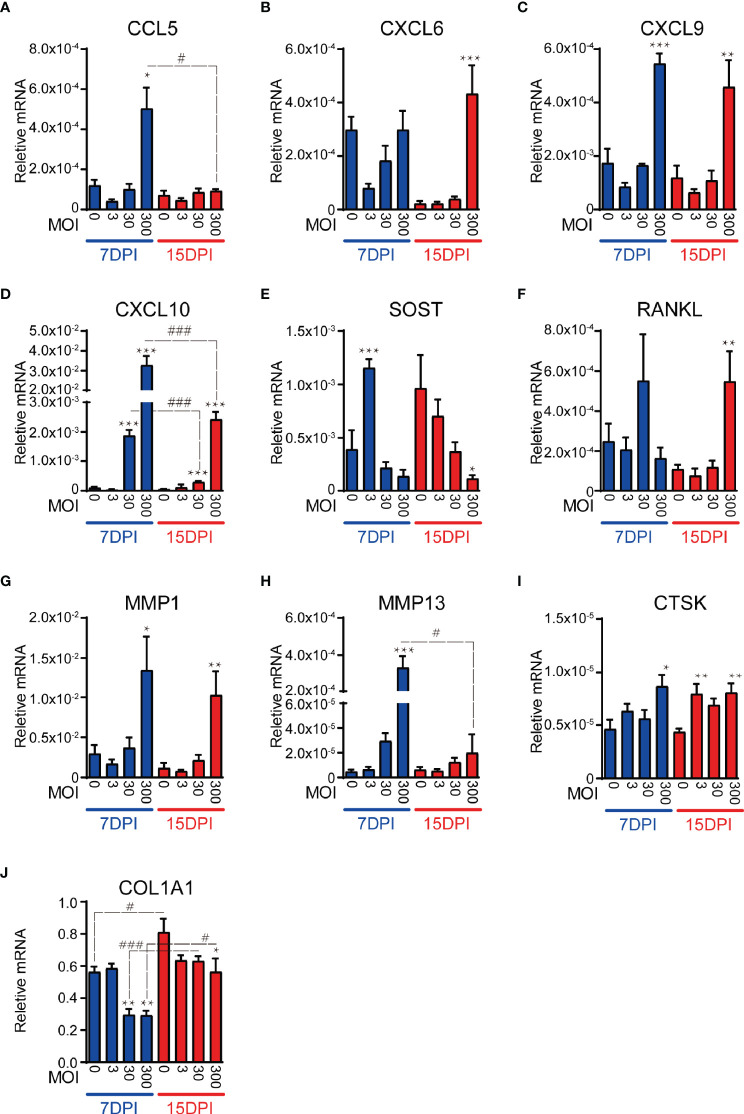
SaOS2-OY gene expression modulation in response to a logarithmic dose range of WCH-SK2 infection over time. Gene expression was normalised to that of the housekeeping gene *ACTB*. Genes tested were either immunomodulatory **(A–D)**, osteocytic markers and mediators of bone turnover **(E, F)** or extracellular matrix effectors **(G–J)**. Data shown are means of biological triplicates each measured in duplicate ± standard error of the mean. Significant changes of the treatment group against the uninfected control within the same time point is indicated by *,**,*** signifying *p* < 0.05, 0.01, and 0.001, respectively, in an ANOVA multiple comparison test. Significant changes for the same MOI at different time points is indicated by ^#^,^##^, ^###^ signifying *p* < 0.05, 0.01, and 0.001, respectively. MOI, multiplicity of infection; DPI, days post-infection.

*CCL5* was up-regulated by infection at 7 DPI although only at the highest MOI. This response returned to basal levels by 15 DPI (**Figure 3A**). *CXCL6* was only upregulated at 15 DPI and in response to the highest MOI (**Figure 3B**). *CXCL9* maintained a strong response to the highest MOI across both time points (**Figure 3C**). *CXCL10* expression displayed a clear dose-dependent effect throughout the 15d period, with a significant and proportionally lower response at MOI 30 compared to MOI 300, although with decreased magnitude at the later time point (**Figure 3D**). It is noteworthy that none of the immune-modulatory genes tested showed a significant increase of expression at the low MOI of 3, and indeed trended downwards, suggesting that very low level infections might not trigger innate immune responses in osteocytes rendering them sub-clinical.

In addition to testing immunomodulatory genes, we examined the expression of osteocyte marker genes. *SOST*, encoding sclerostin– an antagonist of the osteogenic canonical Wnt pathway (Schaffler et al., 2014; Prideaux et al., 2016) and a potent regulator of bone remodelling *via* effects on the osteocyte (Atkins et al., 2011; Wijenayaka et al., 2011), was upregulated at 7 DPI at the lowest MOI tested, which returned to baseline levels at higher MOIs and finally, by 15 DPI there was a significant and dose-dependent decrease in *SOST* expression (**Figure 3E**). *RANKL*, whose expression primarily by osteocytes drives osteoclastic differentiation in adult bone (Xiong and O'Brien, 2012), was upregulated in response to the highest MOI tested at 15 DPI (**Figure 3F**).

In addition to the role of osteocytes as orchestrators of bone resorption by osteoclasts, contributing to the bone destruction associated with osteomyelitis (Hofstee et al., 2020), they are also involved directly in the modification of their extracellular matrix (Tsourdi et al., 2018). Our recent findings show that PJI is associated with loss of intact type I collagen in the bone matrix, contributed to by osteocytes (Ormsby et al., 2021). As such, we tested genes involved in the degradation or deposition of bone extracellular matrix, namely *MMP1*, *MMP13* and *CTSK* or *COL1A1*, respectively. *MMP1* and *MMP13*, whose activated protein products are capable of degrading type I collagen, demonstrated significant up-regulation of expression in response to the highest MOI at 7 DPI (**Figures 3G, H** **)**, consistent with our recent findings in PJI bone, experimentally infected human bone and primary osteocyte-like cells (Ormsby et al., 2021). *MMP1* expression remained elevated at 15 DPI, whilst the *MMP13* response decreased over the duration of the infection *CTSK*, which encodes a major collagenase associated most commonly with osteoclastic bone resorption but also with osteocytic osteolysis, was significantly upregulated by infection at both DPIs tested and whilst the effect was only present in the highest MOI tested at 7 DPI, the up-regulation became uniform across all MOI at 15 DPI (**Figure 3I**). In concert with the elevated levels of collagenases in response to infection, *COL1A1*, which encodes the major fibril of type I collagen, was significantly down-regulated across the course of the infection (**Figure 3J**).

### Effect of Clinically-Relevant Antibiotics on Intracellular *S. aureus* Killing

**Figure 4:**
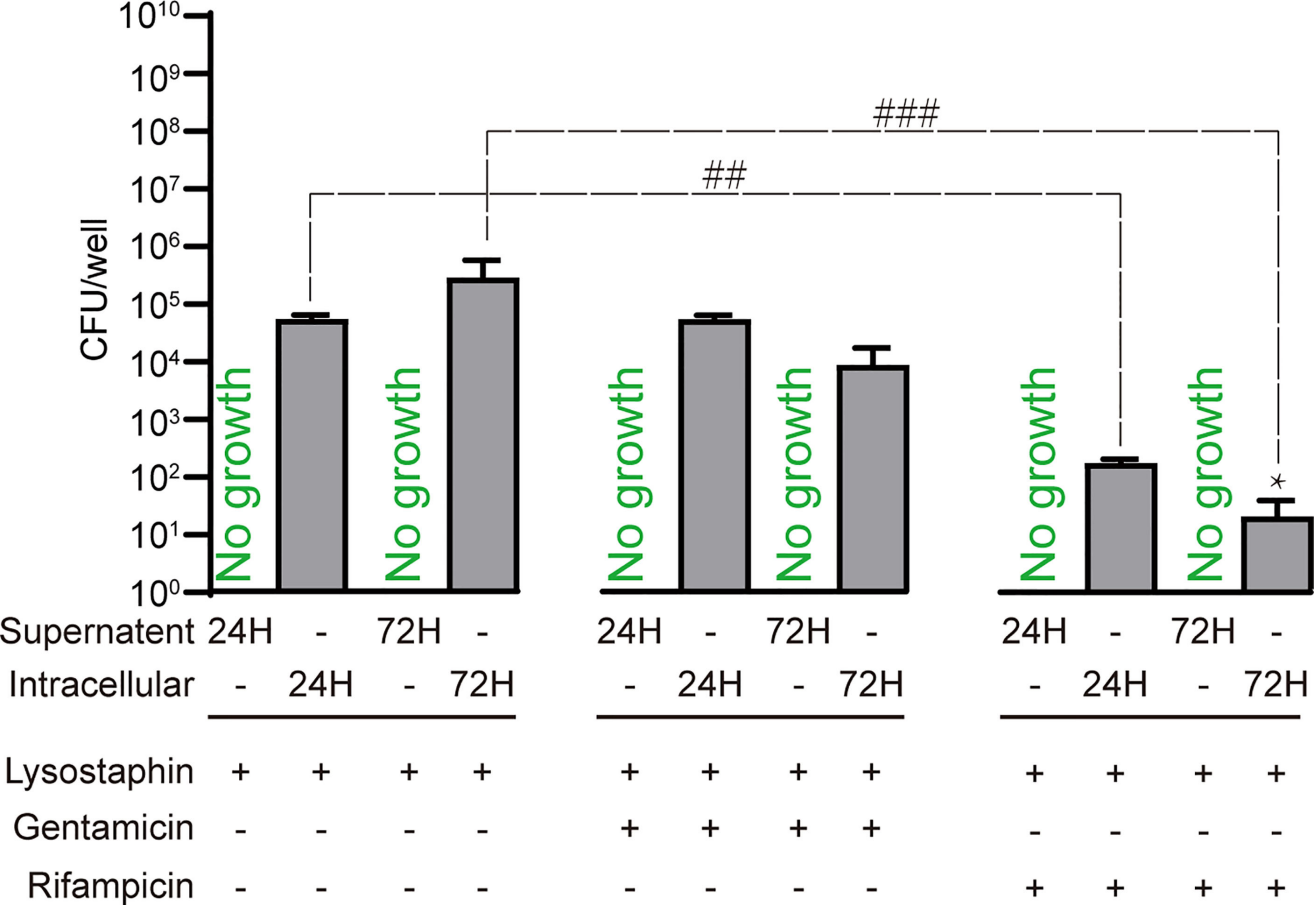
Treatment of SaOS2-OY infected with an MOI of 1 WCH-SK2 with clinically utilised antibiotics. The antibiotic concentrations used were the minimum bactericidal concentration for planktonic growth of this strain, namely 450µg/ml and 0.75µg/ml for gentamicin and rifampicin, respectively. CFU were quantified after 24 and 72 hours of infection from either the lysate to determine bacterial burden or the supernatant to detect potential antibiotic resistance and escape from the intracellular niche. The negative control group was treated with lysostaphin for 2 hours post-infection to clear extracellular bacteria followed by culture in antibiotic free media. Data shown are means of quadruplicate wells ± SD. Significant difference between treatments is indicated by ^##^ *p* < 0.01 and ^###^ *p* < 0.001. Significant difference between time points is indicated by *p < 0.05.

We next sought to use the intracellular infection model to test the ability of clinically utilised antibiotics to clear intracellular *S. aureus*. Infected SaOS2-OY were treated with the minimum bactericidal concentration (MBC) of each antibiotic required to achieve complete killing of the planktonic growth phenotype. Treatment of infected cells with rifampicin was found to cause an over 95% reduction in culturable intracellular bacterial burden at both 24h and 72h, in comparison to the antibiotic-free media controls (**Figures 4**). Conversely, gentamicin had no effect on intracellular bacterial burden at either time point. In all tested cultured wells, we did not find any bacterial out-growth into media, indicating that the bactericidal effects were confined to intracellular bacteria.

## Discussion

The examination of intracellular infection dynamics between SaOS2 before and after differentiation into an osteocyte-like stage strongly supports the biological relevance of this cell line as a model of osteocyte infection. Consistent with our previous work with primary osteoblasts and osteocytes, SaOS2-OY were more capable of both resisting and suppressing infection. Within undifferentiated SaOS2-OB, we observed clusters of apparently replicating bacteria and the escape of bacteria from the intracellular niche, which was likely facilitated in part through the greater cytotoxic response seen in this cell type. This result was consistent with similar work using SaOS2 by Dusane et al., who found that removal of antibiotic pressure resulted in the proliferation and escape of intracellular *S. aureus* (Dusane et al., 2018). In contrast, bacteria within SaOS2-OY did not appear to replicate. A blockade of replication is consistent with the decrease in culturable bacteria over time observed in cell lysates. Bacteria in either host cell type but particularly in SaOS2-OY cells were not co-localised with lysosomes (**Figures 1** and **2**), indicating either inhibition of co-localisation, consistent with previous reports of *S. aureus* blocking autophagosome-lysosome fusion (Schnaith et al., 2007), or that successful lysosomal fusion results in rapid clearance, the latter possibility being supported by the decline in culturable bacteria with time and the observation of bacteria being degraded within SaOS2-OY by TEM. The evidently greater number of lysosomes in SaOS2-OB (**Supplementary Figure S1**) is consistent with their less differentiated state (Prideaux et al., 2014), although this did not appear to assist with suppression of bacterial load. Escape from the intracellular niche was not observed in infected SaOS2-OY cells, unlike the corresponding SaOS2-OB cultures, consistent with the lower cytotoxic effect of infection on osteocytes (Carli et al., 2018). A range of clinical osteomyelitis *S. aureus* isolates were found to cause intracellular infection within SaOS2-OY, although there was a large variation in recovered CFU between these isolates (**Figure 2J**). While we have not explored this further, it is likely attributable to variations in the genetic profiles of the isolates and therefore differences in their ability to be internalised, virulence factor expression, and the relative ability to persist within the host cell environment, for example their relative capability of switching to quasi-dormant lifestyles, such as SCV.

The observed differences in invasiveness and intracellular bacterial dynamics between the osteoblast and osteocyte-like differentiation states can be conceptualised in terms of the respective physiologic niches occupied by these two cell types in bone. As osteoblasts are located on the bone surface they would be more likely to be exposed to greater numbers of bacteria during an infection and as they are a less differentiated cell type with greater proliferative potential, they are less of a metabolic burden to replace following cell death. Localisation at the bone surface also increases the accessibility of immune cells. As such, it is conceivably advantageous to the host for infected osteoblasts to preferentially die rather than risk establishment of a long-lived intracellular bacterial niche. Indeed, it has been reported that undifferentiated SaOS2 cells readily die following intracellular infection (Mohamed et al., 2014). In contrast, bone-encased osteocytes represent a significant metabolic burden to replace *via* bone remodelling and are relatively inaccessible to immune cells (Jilka et al., 2013). Thus, if the host osteocyte dies in response to infection, it is more likely to result in the establishment of an inaccessible biofilm deep within the lacunocanalicular network than beneficial presentation to local professional phagocytic cells, as has been observed *in vivo* (de Mesy Bentley et al., 2017). Long-term dormancy within osteocytes is also more favourable for the pathogen than within osteoblasts, due to the long lifespan of osteocytes and their physical separation from the cell-mediated immune system that functions to detect and eliminate such infections and as such there is the potential for an evolutionary imperative for osteocytes to be better equipped to resist intracellular infection than other bone cell types.

TEM analysis of infected SaOS2-OY revealed evidence for at least some degree of bacterial clearance. Bacteria undergoing degradation were observed to be contained within structures consistent with late autophagolysosomes (Yuan et al., 2012; Hosseini et al., 2014). Large electron lucent gaps surrounding intracellular bacteria were also observed, which are commonly reported to be the structure of phagosomes/autophagosomes prior to lysosomal fusion (Schnaith et al., 2007; Neumann et al., 2016). The observation of bacteria binding to and the invagination of the host cell membrane indicates the existence of the canonical phagocytic pathway of entry into this cell line (Josse et al., 2017). In addition, we observed intact bacteria free within the cytoplasm, indicating the capacity of *S. aureus* to escape from the degradative pathway in osteocytes, consistent with our observations of a lack of co-localisation with lysosomes, perhaps as a prelude to persistence (Moldovan and Fraunholz, 2019).

Analysis of the infection dynamics within SaOS2-OY showed that when *S. aureus* is contained within the intracellular niche, there is a logarithmic decline in culturable bacteria over time, in addition to a transition to a SCV growth phenotype, consistent with our observations in human primary osteocytes (Yang et al., 2018). Time-related enrichment of a quasi-dormant SCV phenotype is consistent with a pathogenic mechanism to adopt a low metabolic and low virulence state that favours long-term intracellular survival. This may decrease the likelihood that the host cell will mount an effective innate immune response to clear the infection or be triggered to undergo apoptosis, both of which would counter this niche of persistence (Ou et al., 2016).

The induction of an SCV growth phenotype was associated with a lower magnitude induction of proinflammatory gene expression. There was reduced expression with time for the majority of the inflammatory chemokines tested. *CCL5* expression most notably was completely abrogated as the infection transitioned into a quasi-dormant state. For example, *CXCL10* expression in response to the highest MOI tested dropped from 307-fold to 45-fold higher than uninfected cells over the experimental time course. This decline would presumably reduce the ability of the infected osteocyte to initiate a sustained immunogenic phenotype, as CXCL10 functions through its receptor CXCR3, which is predominantly expressed by activated T cells, in addition to a high proportion of circulating B, T and natural killer cells (Qin et al., 1998). Additionally, the elevated expression of CXCL10 could play a role in the bone resorption phenotype typical of PJI by promoting osteoclastogenesis (Kwak et al., 2008). CXCL9 is also part of the CXCR3 family of chemokines and shares functional overlap with CXCL10. Whilst the expression of both genes was found to be significantly induced by WCH-SK2 infection, the magnitude of modulation and dose-dependency was notably different. This is consistent with observations in other cell types, where CXCL9 was predominantly expressed by the immune infiltrate and not the local tissue at the site of inflammation (Flier et al., 2001). *CXCL6* was upregulated in response to infection only at the 15 DPI time point, consistent with its role in chronic inflammation (Gijsbers et al., 2004).

The expression changes of the bone matrix modifying factors tested were consistent with osteomyelitic osteolysis. All three collagenases (*MMP1*, *MMP13* and *CTSK*) tested demonstrated increased expression in response to infection. Similarly, a reduction in *COL1A1* expression was seen, together suggesting both a reduction in matrix formation and increased osteocyte-mediated matrix degradation, consistent with our recent observations in PJI patient bone, *ex vivo* human bone and primary osteocyte models of infection (Ormsby et al., 2021). This transition to a catabolic state is also supported by the increased expression of RANKL within late and high dose infections, which would confer an increased rate of osteoclast differentiation at the bone surface.

Finally, the intracellular niche of persistence is associated with antimicrobial resistance (Proctor et al., 2006; Nguyen et al., 2009), as discussed above. As a proof-of-concept, we confirmed the relative resistance of intra-osteocytic *S. aureus* to the clinically utilised antibiotics rifampicin and gentamicin at their respective MBCs for the infective MRSA strain in the planktonic growth state. Whilst rifampicin, which can passively diffuse across the plasma membrane, did cause a significant reduction in recoverable intracellular CFU, an appreciable number of culturable bacteria remained (**Figure 4**). As expected, gentamicin, which is not cell membrane permeable, did not cause a reduction in recoverable CFU. These findings serve to illustrate the point that new antimicrobial strategies need to be designed for the clearance of chronic osteomyelitis where the potential for intracellular infection exists.

In summary, SaOS2-OY cells are a useful and biologically relevant human osteocyte-like cell line model, with which to explore mechanisms of *S. aureus* intracellular persistence in the context of infectious osteomyelitis. This model offers ease of availability over primary cell models and intra-assay consistency independent from human donor variation. As a human cell line, the model may also provide findings of closer immediate clinical relevance than the extant non-human *in vitro* models. Further, it may prove suitable for high throughput applications, such as screening candidate antimicrobial treatments against intracellular infections.

## Data Availability Statement

The original contributions presented in the study are included in the article/**Supplementary Material**. Further inquiries can be directed to the corresponding authors.

## Author Contributions

All authors contributed to conception and design of the study. NG and AZ performed experiments and the statistical analysis. GA and SK provided resources for the experiments. NG wrote the first draft of the manuscript. DY, GA, and AZ wrote sections of the manuscript. All authors contributed to manuscript revision, read, and approved the submitted version.

## Conflict of Interest

The authors declare that the research was conducted in the absence of any commercial or financial relationships that could be construed as a potential conflict of interest.

## Publisher’s Note

All claims expressed in this article are solely those of the authors and do not necessarily represent those of their affiliated organizations, or those of the publisher, the editors and the reviewers. Any product that may be evaluated in this article, or claim that may be made by its manufacturer, is not guaranteed or endorsed by the publisher.
