# Supplementary material for: A Human Osteocyte Cell Line Model for Studying Staphylococcus aureus Persistence in Osteomyelitis
Source: Front Cell Infect Microbiol. 2021 Nov 3;11:781022. doi: 10.3389/fcimb.2021.781022 (PMC8597899; doi:10.3389/fcimb.2021.781022)
Supplement: Supplementary file 1 [file Presentation_1.pptx]

## Slide 1
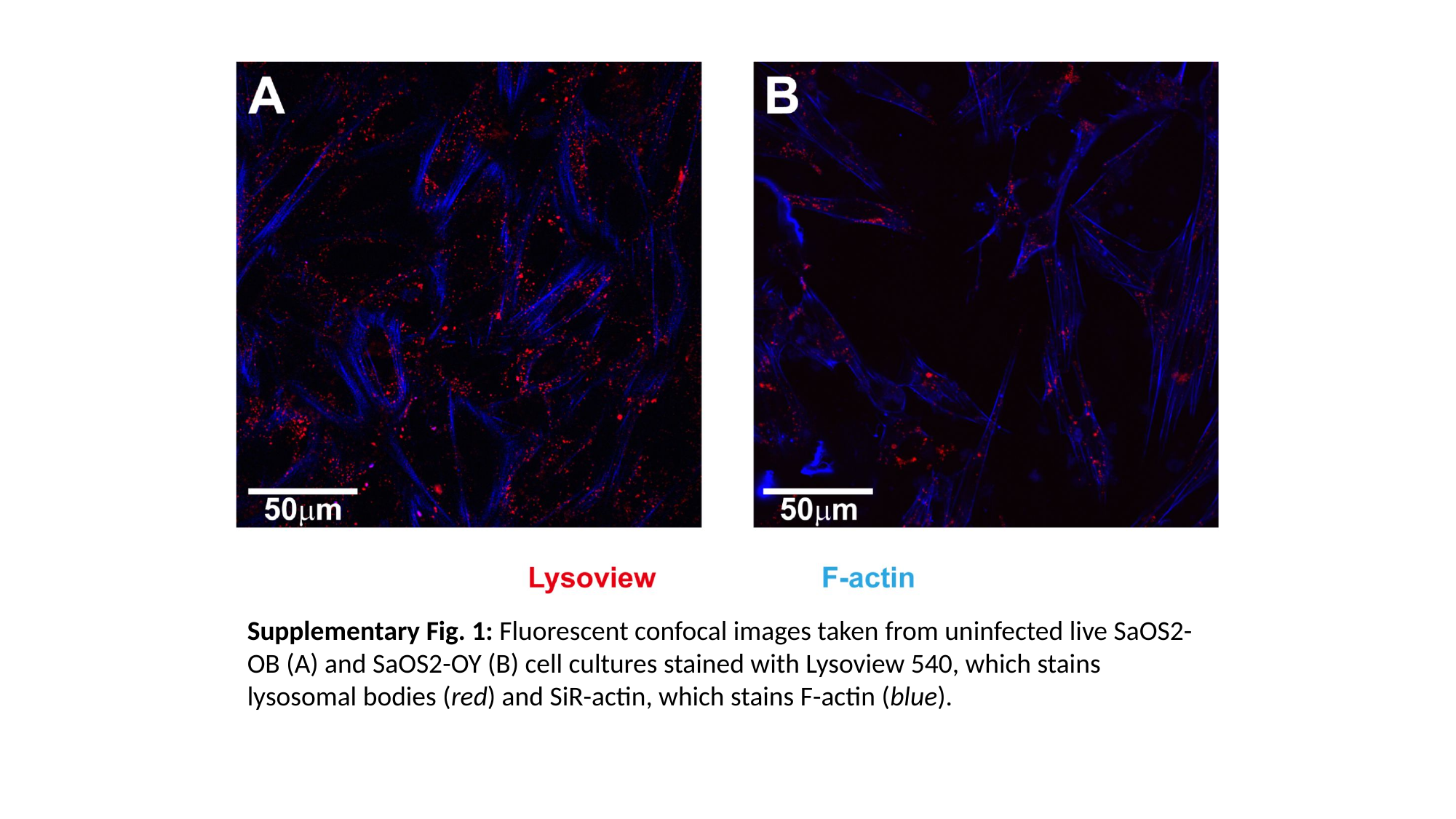

Supplementary Fig. 1: Fluorescent confocal images taken from uninfected live SaOS2-OB (A) and SaOS2-OY (B) cell cultures stained with Lysoview 540, which stains lysosomal bodies (red) and SiR-actin, which stains F-actin (blue).
