# Supplementary material for: A Human Osteocyte Cell Line Model for Studying Staphylococcus aureus Persistence in Osteomyelitis
Source: Front Cell Infect Microbiol. 2021 Nov 3;11:781022. doi: 10.3389/fcimb.2021.781022 (PMC8597899; doi:10.3389/fcimb.2021.781022)
Supplement: Supplementary file 2 [file Table_1.docx]

**TABLE S1:** RT-PCR primer sets used in this study.

| *Gene name* | *Forward primer* | *Reverse primer* | *Amplicon size (bp)* |
| --- | --- | --- | --- |
| *CCL5* | gtgctccaacccagcagtc | catcctagctcatctccaaag | 113 |
| *CXCL6* | tcatccagaaaattttggacag | atctccagaaaactgctccg | 104 |
| *CXCL9* | gagtgcaaggaaccccagtag | agggcttggggcaaattgtt | 113 |
| *CXCL10* | gtccacgtgttgagatcattgc | atcgattttgctcccctctg | 151 |
| *RANKL* | tcagccttttgctcatctcactat | ccacccccgatcatggt | 96 |
| *SOST* | accacccctttgagaccaaag | ggtcacgtagcgggtgaagt | 79 |
| *MMP1* | aaacacatctgacctacagga | ttactccagagttggaaggc | 97 |
| *MMP13* | ggatccagtctctctatggt | ggcatcaagggataaggaag | 95 |
| *CTSK* | ggccaactcaagaagaaaactg | tctctgtaccctctgcatttagc | 230 |
| *COL1A1* | agggctccaacgagatcgagatccg | tacaggaagcagacagggccaacgtcg | 225 |
| *ACTB* | aagagatggccacggct | caatgatcttgatcttcattgtgc | 318 |
